# Campylobacter jejuni Survives within Epithelial Cells by Avoiding Delivery to Lysosomes

**DOI:** 10.1371/journal.ppat.0040014

**Published:** 2008-01-25

**Authors:** Robert O Watson, Jorge E Galán

**Affiliations:** Section of Microbial Pathogenesis, Yale University, School of Medicine, New Haven, Connecticut, United States of America; Johns Hopkins School of Medicine, United States of America

## Abstract

Campylobacter jejuni is one of the major causes of infectious diarrhea world-wide, although relatively little is know about its mechanisms of pathogenicity. This bacterium can gain entry into intestinal epithelial cells, which is thought to be important for its ability to persistently infect and cause disease. We found that C. jejuni is able to survive within intestinal epithelial cells. However, recovery of intracellular bacteria required pre-culturing under oxygen-limiting conditions, suggesting that C. jejuni undergoes significant physiological changes within the intracellular environment. We also found that in epithelial cells the C. jejuni–containing vacuole deviates from the canonical endocytic pathway immediately after a unique caveolae-dependent entry pathway, thus avoiding delivery into lysosomes. In contrast, in macrophages, C. jejuni is delivered to lysosomes and consequently is rapidly killed. Taken together, these studies indicate that C. jejuni has evolved specific adaptations to survive within host cells.

## Author Summary

Campylobacter jejuni is one of the most common causes of food-borne illness in the United States and a major cause of diarrheal disease throughout the world. After infection through the oral route, this bacterium invades the cells of the intestinal epithelium, a property that is important for its ability to cause disease. Usually, bacteria and other material entering the cell move to compartments called lysosomes, where an acidic mix of enzymes breaks it down. This study shows that C. jejuni can survive within intestinal epithelial cells by avoiding delivery to lysosomes. In contrast, in macrophages, which are specialized cells with the capacity to engulf and kill bacteria, C. jejuni cannot avoid delivery into lysosomes and consequently is rapidly killed. These studies help explain an important virulence attribute of C. jejuni.

## Introduction

Campylobacter jejuni is the leading cause of bacterial food-borne illness in the United States and a major cause of diarrheal disease throughout the world [1]. C. jejuni infection is also an important pre-condition for Guillain-Barré paralysis [2]. Despite its public health importance, relatively little is known about its pathogenesis. Examination of intestinal biopsies of humans [3], in vivo studies in infected primates [4] and other animal models [5–7], together with in vitro experiments using cultured human intestinal epithelial cells [8–10], have demonstrated that C. jejuni can invade non-phagocytic intestinal epithelial cells. However, to date, little is known about the molecular details of the mechanisms by which C. jejuni enters intestinal epithelial cells. Bacterial factors such as motility, glycosylation, and capsular synthesis have been implicated in C. jejuni internalization [11–14]. Strains with mutations in these pathways have defects in their ability to adhere to and invade host cells, as well as to colonize animals [12–19]. Bacterial invasion has also been correlated with C. jejuni's ability to stimulate the activation of MAP kinases leading to the production of the pro-inflammatory cytokine, IL-8 [20,21]. Taken together, these data suggest that bacterial internalization into intestinal epithelial cells is important in C. jejuni pathogenesis.

Although most host factors that are required for C. jejuni internalization into non-phagocytic cells remain unknown, this entry process appears to have unique cytoskeletal requirements. Most other bacterial pathogens such as Listeria monocytogenes, Shigella flexneri, and Salmonella typhimurium utilize the host-cell actin cytoskeleton to gain intracellular access [22]. However, C. jejuni is internalized into intestinal epithelial cells in a microtubule-dependent, actin-independent fashion [10], suggesting that this bacterium employs an entry mechanism unlike those reported for other bacterial pathogens.

The intracellular fate of C. jejuni remains unknown, although it is likely that this bacterium, similar to other intracellular pathogens, may have evolved specific adaptations to survive within host cells. Intracellular pathogens utilize a variety of strategies to survive and replicate within host cells. For example, some pathogens such as Trypanasoma cruzi [23], Listeria monocytogenes [24], and Shigella flexneri [22,25] break out of the phagocytic vacuoles after internalization and can replicate within the cytosol of the infected cell. Other pathogens, such as *Leishmania*, have evolved an array of adaptations to survive in the hostile environment of the phagolysosome, which is characterized by low oxygen tension, poor nutrient content, low pH, and the presence of a variety of antibacterial products such as antibacterial peptides and lysosomal enzymes [26]. Yet another group of intracellular pathogens survive within a vesicular compartment that does not fuse with lysosomes. For example, Salmonella typhimurium [27] and Mycobacterium tuberculosis [28] alter the biogenesis and dynamics of their vacuolar compartment preventing fusion to lysosomes.

All evidence to date indicates that after internalization into intestinal epithelial cells, C. jejuni resides within a membrane bound compartment [29–31]. We report here that C. jejuni survives within intestinal epithelial cells by deviating from the canonical endocytic pathway thus residing in a unique intracellular compartment that does not fuse with lysosomes.

## Results

### Campylobacter jejuni Survives within Intestinal Epithelial Cells but Not within Bone Marrow–Derived Macrophages

**Figure 1:**
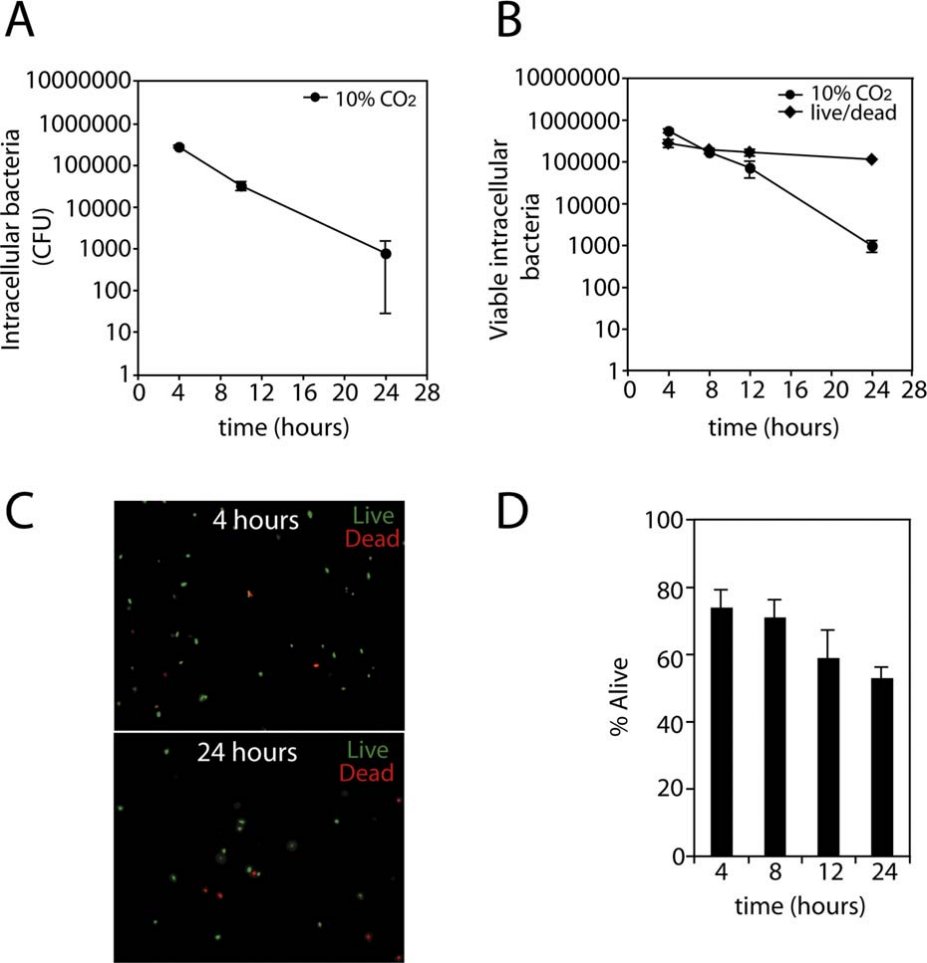
Survival of C. jejuni within Cultured Intestinal Epithelial Cells Human intestinal epithelial T84 cells were infected with C. jejuni and viable intracellular bacteria were assessed over time by CFU recovery (A and B) or live/dead staining (B–D). (A) Viable CFU of intracellular C. jejuni were determined at the indicated times after gentamicin treatment. (B) Viable intracellular C. jejuni was analyzed at the indicated times via CFU recovery as in A or live/dead staining and FACS analysis as described in the Materials and Methods. (C) Epiflourescent visualization of a representative field of intracellular C. jejuni isolated at 4 h and 24 h post-infection and subjected to live (green)/dead (red) staining. (D) Intracellular C. jejuni was isolated at different times after infection and the number of viable and non-viable intracellular bacteria was quantified via live/dead staining and FACS analysis. Values are expressed as percent of live bacteria. In all cases, values are the mean and standard deviation of three independent experiments.

**Figure 2:**
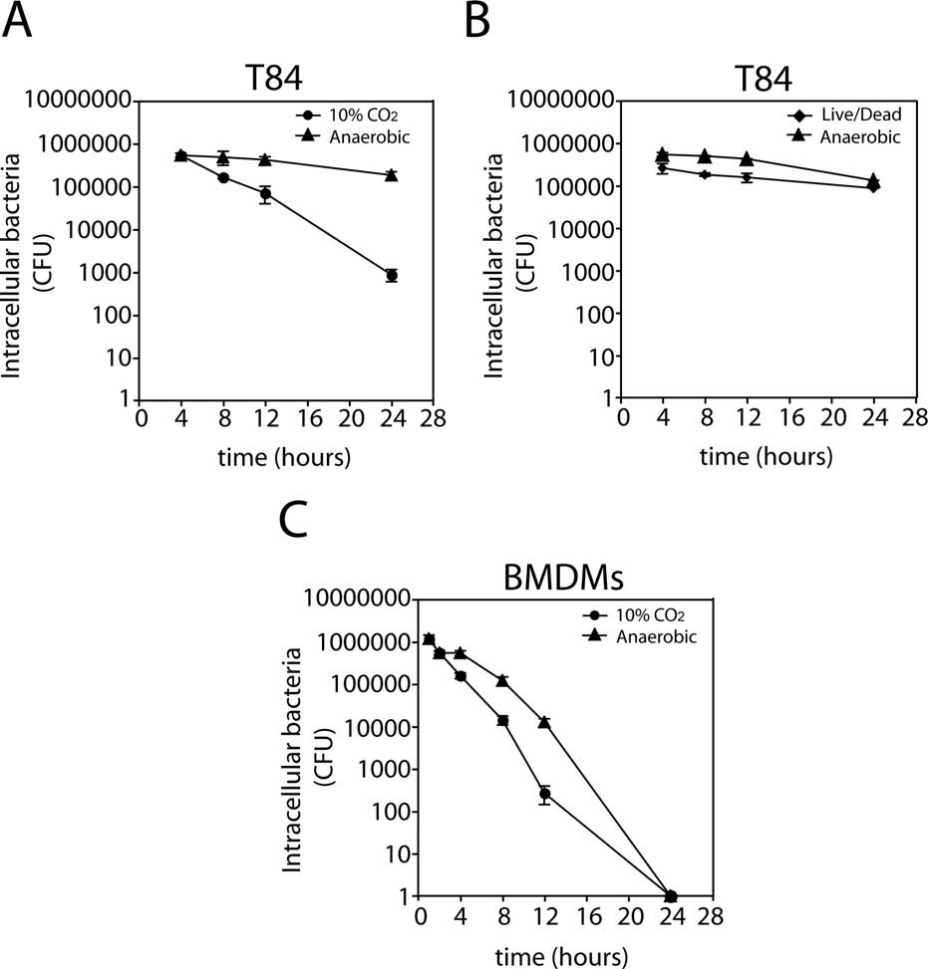
C. jejuni Remains Viable within Intestinal Epithelial Cells but Does Not Survive within Bone Marrow–Derived Macrophages (A) Human intestinal epithelial T84 cells were infected with C. jejuni, and viable intracellular bacteria were assessed over time by CFU recovery under 10% CO_2_ or oxygen-limiting conditions as described in Materials and Methods. (B) The number of viable intracellular bacteria was analyzed in parallel via CFU recovery plated under oxygen-limiting conditions or live/dead staining coupled with FACS analysis. (C) Mouse bone marrow–derived macrophages were infected with C. jejuni and viable bacteria CFU were determined over time after 10% CO_2_ or oxygen-limiting cultivation. All values are the mean and standard deviation of three independent determinations.

Although C. jejuni internalization into host cells is believed to play a role in pathogenesis, little is known about its intracellular fate. We therefore examined the ability of C. jejuni to survive within intestinal epithelial cells. Human intestinal epithelial T84 cells were infected with C. jejuni, and total viable intracellular bacteria were determined at different times by counting colony forming units (CFU). Significant numbers of CFU (> 3 × 10^5^/well) were recovered at early time points, however, over time, the number of CFU recovered decreased considerably (Figure 1A). By 24 h there was a ∼500-fold decrease in the number of CFU recovered from infected cells compared to 4 h after infection (Figure 1A). These results suggest that intracellular C. jejuni rapidly loses viability during the course of its intracellular stage. This was surprising as it suggested that the ability of C. jejuni to enter non-phagocytic cells might not confer a significant advantage to this bacterium. We therefore examined the possibility that internalized C. jejuni may alter its physiology in such a way that, although viable, it may not be culturable under the conditions used in this assay. Indeed, C. jejuni has been reported to enter a viable but non-culturable state when subjected to a variety of stimuli or environments [32–34]. To address this issue, we stained C. jejuni recovered from cultured intestinal T84 cells with reagents that distinguish viable from non-viable bacteria (see Materials and Methods). Using these reagents, we observed no decrease in viability of intracellular C. jejuni over time (Figure 1B and 1C). In fact, FACS analysis also revealed that the ratio of viable to non-viable bacteria did not change over the course of infection (Figure 1C and 1D). These results indicate that C. jejuni remains viable for at least 24 h after infection and suggest that it acquires a physiological state that does not allow the recovery of CFU under our standard culture conditions. We hypothesized that once internalized by intestinal epithelial cells, C. jejuni might adapt to the low oxygen environment within the cell by changing its mode of respiration. We therefore tested whether the intracellular bacterial population could be cultured if allowed to “recover” under conditions in which oxygen is very limiting. Human intestinal epithelial T84 cells were infected with C. jejuni and the number of CFU was determined after culturing under oxygen-limiting incubation or under 10% CO_2_ conditions. The number of CFU decreased drastically (∼500 fold) when bacteria were directly grown under an atmosphere of 10% CO_2_ (Figure 2A) or in GasPak jars (BBL Microbiology Systems, Cockeysville, MD) with BBL CampyPacks (BBL Microbiology Systems, Cockeysville, MD) (data not shown). However, there was no significant decrease in the number of CFU recovered over time when the plates were incubated under oxygen-limiting conditions for 48 h and then switched to an atmosphere of 10% CO_2_ (Figure 2A). The number of CFU recovered when plates where pre-incubated under low-oxygen conditions closely correlated with the number of viable bacteria quantified via FACS analysis (Figure 2B). These data demonstrate that C. jejuni remains viable within intestinal epithelial cells for at least 24 h, although it undergoes physiological changes such that requires exposure to oxygen-limiting conditions for its efficient recovery.

There are contradictory reports regarding the ability of C. jejuni to survive within macrophages [29,35–38]. Given our findings in intestinal epithelial cells suggesting physiological changes in intracellular C. jejuni, which affects its ability to be cultured, we re-examined its ability to survive within macrophages. Mouse primary bone marrow–derived macrophages (BMDM) were infected with C. jejuni and the number of CFU recovered over time was determined by incubating culture plates directly under either 10% CO_2_ or subjecting them to a 48 h pre-incubation under oxygen-limiting conditions prior to incubation under 10% CO_2_. We found a significant decrease in the number of CFU recovered over time regardless of the culture condition. By 12 h there was a severe reduction in the number of CFU recovered under both 10% CO_2_ or with pre-incubation under oxygen-limiting conditions (>2,000 and >100 fold, respectively, compared to the CFU recovered 1 h after infection) (Figure 2C), and no CFU were recovered at 24 h of infection regardless of the culture conditions. Taken together, these data demonstrate that intracellular C. jejuni can survive within intestinal epithelial cells but are killed by professional phagocytes.

### C. jejuni Avoids Delivery to Lysosomes in Epithelial Cells

**Figure 3:**
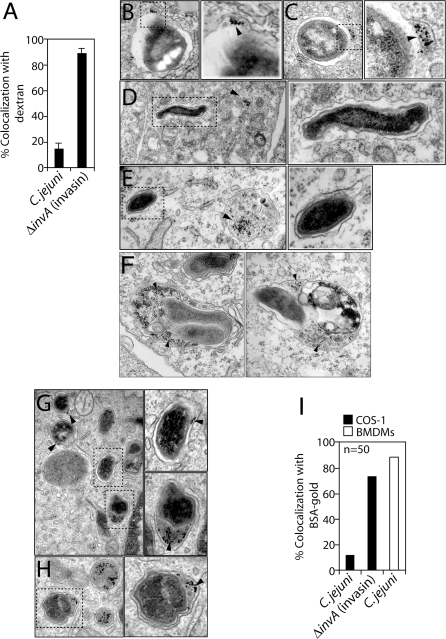
The C. jejuni–Containing Vacuole Is Accessible to Endocytic Tracers in Macrophages but Not in Epithelial Cells (A) Cos-1 cells were infected with *S. typhimurium invA* (invasin) expressing the dsRed protein or *C. jejuni*, pulsed with the endocytic tracers dextran to label lysosomes, and phagosomes containing bacteria that colocalized with endocytic tracer were quantified. Results are the means and standard deviation of three independent experiments. For each experiment, at least 100 phagosomes were counted. Alternatively, infected cells were pulsed with the endocytic tracer BSA-gold and visualized by electron microscopy. Electron micrographs of phagosomes containing *S. typhimurium invA* (invasin) (B and C) or C. jejuni (D, E and F) are shown. C. jejuni–containing vacuoles (CCVs) that do not colocalize with BSA-gold (D and E) as well CCVs that colocalize with BSA-Gold and resemble lysosomes (F) are shown for comparison (see text). In addition, bone marrow–derived macrophages were infected with C. jejuni and pulsed with BSA-gold and visualized by electron microscopy. Electron micrographs of phagosomes containing C. jejuni are shown (G and H). Arrows indicate BSA-gold. Higher magnifications of the indicated insets are also shown to the right of each electron micrograph. Quantification of the colocalization of BSA-gold with bacterial vacuoles in the different cells are shown (I). Results are representative of two independent experiments in which at least 50 vacuoles were counted for colocalization with BSA-gold.

Immediately after internalization into host cells, C. jejuni resides within a membrane bound compartment [29–31]. It is therefore possible that in order to survive within cells, this bacterium has evolved specific adaptations to survive within lysosomes or to modulate host cellular trafficking events to avoid fusion with lysosomes. If C. jejuni survives within lysosomes, the C. jejuni–containing vacuole (CCV) should be accessible to endocytic tracers. To test this hypothesis, Cos-1 cells were first infected with C. jejuni and subsequently exposed to the fluorescent endocytic tracer dextran, which was chased into lysosomes. As a control, cells were infected with a strain of S. typhimurium carrying a mutation in *invA* and a plasmid expressing the Yersinia pseudotuberculosis protein invasin. InvA is an essential component of the invasion-associated type III secretion system [39] and therefore this strain enters cells through the invasin-mediated pathway [40]. Subsequent to its uptake, the invasin-expressing bacteria is delivered to lysosomes (Watson and Galán, unpublished data). Immunofluorescense microscopy analysis revealed that 90% of the vacuoles containing *S. typhimurium invA* (invasin) colocalized with the endocytic tracer dextran (Figure 3A and Figure S1). In contrast, only ∼15% of the C. jejuni–containing vacuoles acquired detectible amounts of dextran (Figure 3A and Figure S1). These data suggest that the CCV is functionally separated from the canonical endocytic pathway. To confirm these results, we used another endocytic tracer, gold-labeled bovine serum albumin (BSA-gold) [41]. Cos-1 cells were first infected with C. jejuni or *S. typhimurium invA* (invasin) and subsequently exposed to BSA-gold, which was chased into lysosomes (see Materials and Methods). BSA-gold was then imaged using electron microscopy to determine if the CCV was accessible to this fluid phase endocytic tracer. Although BSA-gold colocalized with ∼75% of the vacuoles containing *S. typhimurium invA* (invasin) (Figure 3B, 3C and 3I), only 15% of the CCVs were accessible to the endocytic marker (Figure 3D, 3E and 3I). Furthermore, the CCVs that colocalized with BSA-gold appeared to be morphologically different from the CCVs that did not. The CCVs accessible to the endocytic tracer were spacious and contained additional electron dense materials, closely resembling lysosomes (Figure 3F). In contrast, the CCVs that did not colocalize with BSA gold had tight membranes around the bacteria and the compartments did not resemble lysosomes (Figure 3D and 3E). These data confirm the results obtained using fluorescence microscopy and provide additional evidence that the CCV is segregated from the canonical endocytic pathway.

Since our experiments established that C. jejuni quickly loses viability within macrophages, we hypothesized that in these cells this bacterium may be delivered to lysosomes. To test this hypothesis, BMDM were infected with C. jejuni and then incubated with media containing BSA-gold and examined by electron microscopy as described in Materials and Methods. In contrast to what we observed in epithelial cells, in macrophages over 90% of the CCVs were readily accessible to the endocytic tracer (Figure 3G, 3H and 3I). Similar results were obtained using fluorescent dextran as an endocytic tracer (data not shown). Taken together, these data indicate that in macrophages, C. jejuni is delivered to lysosomes where it cannot survive, while in intestinal epithelial cells C. jejuni is segregated from an endocytic pathway leading to lysosomes and consequently survives in a vacuolar compartment that is distinct from lysosomes.

### Avoidance of Its Delivery to Lysosomes Is Essential for C. jejuni's Ability to Survive Intracellularly

**Figure 4:**
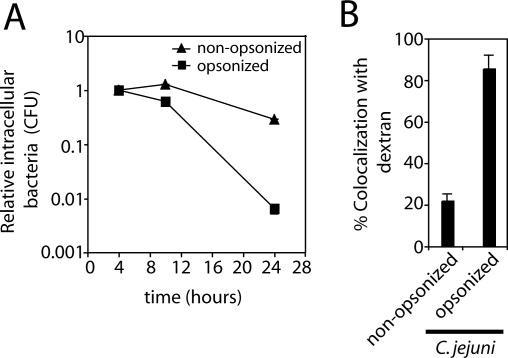
C. jejuni Does Not Survive within Epithelial Cell Lysosomes (A) Cos-1 cells were transfected with a plasmid expressing the Fc-receptor. Twenty-four hours after transfection, cells were infected with opsonized or non-opsonized C. jejuni and bacterial loads quantified over time by culturing under oxygen-limiting permissive conditions. Values are the mean and standard deviation of three independent experiments and are expressed as relative CFU where the number of CFU at each time point was normalized to the initial number of internalized bacteria, which was considered to be 1. (B) The accessibility of the C. jejuni–containing vacuole to the endocytic tracer in transfected cells six hours after infection was assessed by enumeration of colocalization of C. jejuni with dextran as described in Materials and Methods. Results are the means and standard deviation of three independent experiments.

We next examined whether avoidance of lysosomal delivery was essential for C. jejuni survival within epithelial cells. To this end, we carried out an experiment in which C. jejuni was internalized via the Fc receptor, a pathway known to lead to lysosomes [42]. Cos-1 cells expressing the murine Fc receptor were infected with either opsonized or non-opsonized C. jejuni and at different times after infection the CFU were determined by plating under the permissive oxygen-limiting conditions and further incubation in 10% CO2 environment. As shown in Figure 4A, the relative number of CFU recovered from cells infected with opsonized C. jejuni 24 h after infection was significantly (> 20 fold) lower than the number of CFU recovered from cells infected with non-opsonized bacteria. These results indicate that internalization via the Fc receptor results in a significant loss of intracellular viability, presumably because these bacteria are ultimately delivered to lysosomes. To confirm this hypothesis, we examined whether C. jejuni internalized via the Fc receptor was accessible to an endocytic tracer. Cos-1 cells expressing the mouse Fc receptor were infected with opsonized C. jejuni and subsequently exposed to fluorescent dextran. Consistent with the hypothesis that the loss of viability of C. jejuni internalized via the Fc receptor was due to its delivery to lysosomes, >80% of the opsonized bacteria colocalized with fluorescent dextran, compared to ∼20% of non-opsonized control (Figure 4B). Taken together, these data show that C. jejuni is unable to survive within lysosomes and further indicate that this bacterium has evolved a mechanism to avoid delivery to this compartment in order to survive within intestinal epithelial cells. Furthermore, these results also indicate that the mechanism of bacterial entry into host cells has a major impact in the ability of C. jejuni to survive intracellularly.

### Acquisition of Endocytic Markers by the C. jejuni–Containing Vacuole

**Figure 5:**
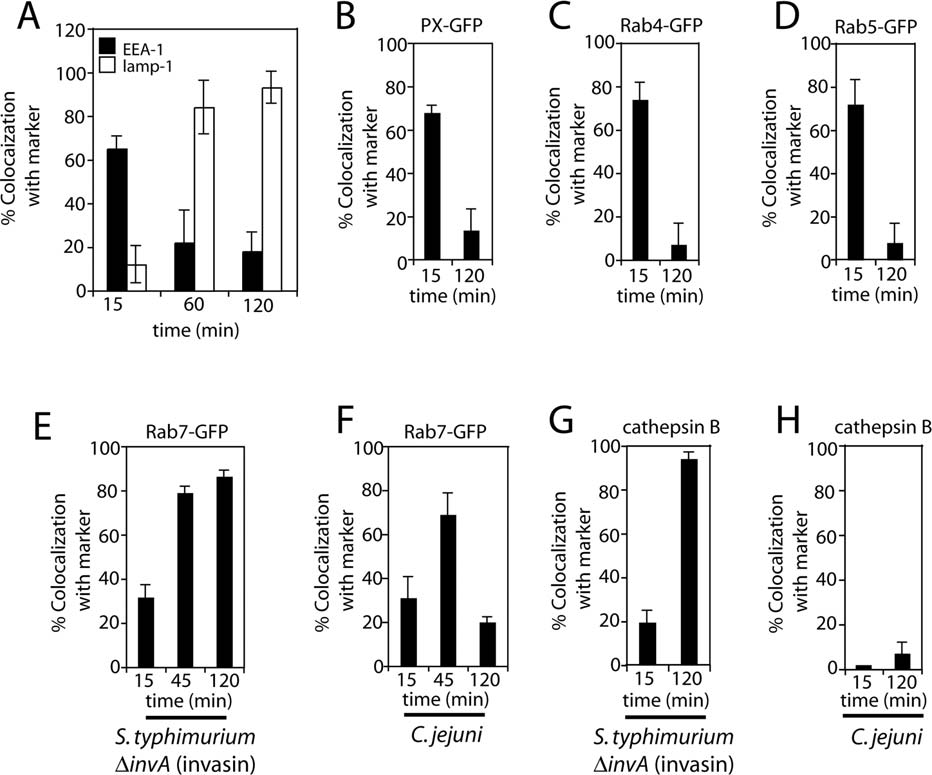
The C. jejuni–Containing Vacuole Acquires Markers of the Early and Late Endocytic Pathway but Not of Lysosomes Cos-1 cells were infected with C. jejuni and or *S. typhimurium invA* (invasin), as indicated. At the designated times, cells were fixed and processed for immunofluorescence using antibodies directed to C. jejuni or S. typhimurium and to the endocytic markers EEA-1 and lamp-1 (A and B), or cathepsin B (G and H). Alternatively, Cos-1 cells were transfected with plasmids expressing PX-GFP (a probe for phosphoinositide 3 phosphate) (C), Rab4-GFP (D), Rab5-GFP (E), or Rab7-GFP (F) , and subsequently infected with C. jejuni or *S. typhimurium invA* (invasin) as indicated. Cells were fixed and processed for immunofluorescence using antibodies directed to C. jejuni or S. typhimurium. At the indicated times, the number of bacteria*-*containing vacuoles that colocalized with the different markers was quantified by immunofluorescence microscopy as indicated in Materials and Methods. Results are the means and standard deviation of three independent experiments. For each experiment at least 100 vacuoles were counted for acquisition of each marker.

To investigate the biogenesis and trafficking of the CCV, we examined the dynamics of acquisition of both early and late endosomal markers. Cos-1 cells were infected as described in Materials and Methods and at different times after infection the presence of different endocytic markers was probed by immunofluorescence microscopy using specific antibodies. Fifteen minutes after infection the majority (∼65%) of the intracellular bacteria co-localized with the early endosomal marker EEA-1 (Figure 5A and Figure S2). However, by 60 min, only ∼20% of the CCV co-localized with EEA-1 and more than 80% were stained by an antibody directed to the late endosomal marker lamp-1 (Figure 5B and Figure S2). Two hours after infection, almost all CCVs stained with lamp-1. The acquisition of lamp-1 may therefore occur via an alternative pathway since, as shown above, the CCV does not fuse with lysosomes.

In an effort to better understand the nature of the C. jejuni compartment, we tested the CCV for the presence of other markers of the early and late endocytic pathway. We found that early in infection, 65–70% of the CCVs co-localized with the early endosomal markers Rab4, Rab5, and with a probe for phosphoinisitide 3 phosphate (green fluorescent protein fused to the PX domain of the 40 kD subunit of the nicotinamide adenine nucleotide phosphate oxidase) (Figure 5B-5D, Figure S3 and Video S1). Two hours after infection, less than 10% of the CCVs colocalized with any of these markers indicating that the CCV interacts transiently with these compartments (Figure 5B-5D). Furthermore, C. jejuni transiently acquires the late endosomal marker Rab7 (Figure 5E, 5F and Figure S4). At 45 min after infection, ∼65% of CCVs acquired Rab7-GFP, while at 2 h, only ∼20% of CCVs colocalized with Rab7-GFP. However, consistent with the observation that the mature CCV does not co-localize with endocytic tracers, the lysosomal marker cathepsin B was seen in only a very small proportion of the CCVs, even at 2 h after infection (Figure 5H and Figure S4), although it was present in 95% of vacuoles containing *S. tyhimurium invA* (invasin) (Figure 5G and Figure S4). These data further indicate that C. jejuni survives within a unique intracellular compartment that despite harboring the lamp-1 protein, is functionally distinct from lysosomes.

**Figure 6:**
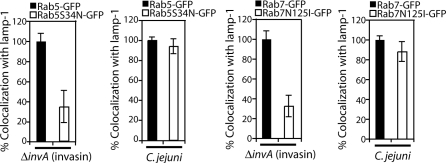
Dominant-Negative Rab5 and Rab7 Do Not Block Acquisition of lamp-1 by the C. jejuni–Containing Vacuole Cos-1 cells were transfected with plasmids expressing either Rab5-GFP, Rab7-GFP, or the dominant negative mutants Rab5^S34N^-GFP or Rab7^N125I^-GFP. Twenty-four hours after transfection, cells were infected with C. jejuni or *S. typhimurium invA* (invasin) for 15 min followed by a 1 h gentamicin chase. Cells were fixed, and lamp-1 acquisition was assessed after processing for immunofluorescence using anti-C. jejuni or anti-S. typhiumurium and lamp-1 antibodies. Results are the means and standard deviation of three independent experiments. For each experiment, at least 100 vacuoles were counted for colocalization with lamp-1.

Our results indicate that at some point after internalization, the CCV deviates from the canonical endocytic pathway. Therefore, we set out to determine at what stage of the endocytic pathway this segregation might occur. The GTPases Rab5 and Rab7 are involved in the biogenesis of early and late endosomes, respectively [43]. Overexpression of dominant negative forms of these GTPases disrupt temporal and spatial delivery of internalized cargo to lysosomes [43]. To determine if acquisition of lamp-1 by the CCV required Rab5 or Rab7, Cos-1 cells were transfected with wild type or dominant negative forms of Rab5 (Rab5^S34N^) or Rab7 (Rab7^N125I^). The transfected cells were infected with C. jejuni and the acquisiton of lamp-1 by the CCV was assessed by immunofluorescence microscopy. As a control, a similar experiment was conducted using *S. typhimurium invA* (invasin), which traffics to lysosomes. Overexpression of Rab5^S34N^ and Rab7^N125I^ did not affect lamp-1 acquisition by the CCV, although it effectively prevented acquisition of this marker by the vacuoles containing *S. typhimurium invA* (invasin) (Figure 6 and Figure S5). These results demonstrate that the CCV acquires lamp-1 by an alternative pathway apparently segregating from the canonical endocytic pathway early after C. jejuni internalization.

### Caveolae and Caveolin-1 Are Required for Efficient C. jejuni Entry into Epithelial Cells

**Figure 7:**
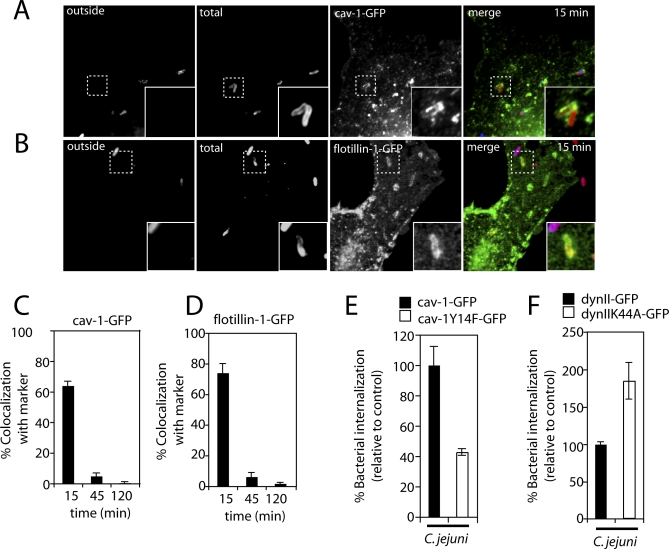
Caveolin Is Recruited to the C. jejuni–Containing Vacuole and Is Required for Efficient C. jejuni Entry Into Epithelial Cells Cos-1 cells were transfected with plasmids expressing caveolin-1-GFP or flotillin-1-GFP. Twenty-four hours after transfection, cells were infected with C. jejuni, fixed at the designated time points, and processed for immunofluorescence using a staining protocol that distinguishes extracellular from internalized bacteria. Images corresponding to 15 min after infection show colocalization of (A) caveolin-1-GFP (green) (A) and flotillin-1-GFP (green) (B) with intracellular C. jejuni (red). The colocalization of C. jejuni with caveolin-1-GFP (C) or flotillin-1-GFP (D) was quantified at the indicated times after infection as described in Materials and Methods. Results are the means and standard deviation of three independent experiments. For each experiment, at least 100 vacuoles were counted for colocalization with each marker for each time point. In addition, Cos-1 cells were transfected with plasmids expressing caveolin-1-GFP, dynamin II-GFP, or their dominant negative mutants caveolin-1^Y14F^-GFP or dynaminII^K44A^-GFP (E and F). Twenty-four hours after transfection, cells were infected with C. jejuni, fixed, internalized bacteria were quantified 3 h after infection as described in the Materials and Methods. The values are the mean and standard deviation of three independent experiments and are expressed as a percentage of the control (caveolin-1-GFP and dynaminII-GFP, respectively), which was considered to be 100%. For each experiment the number of intracellular bacteria was determined for at least 50 transfected cells. The difference between the levels of internalization in cells expressing wild type and dominant-negative mutant forms of caveolin or dynamin were significantly different (unpaired *t*-test *p* = 0.02 for both sets of samples).

Collectively, our data suggest that the mechanism by which C. jejuni enters epithelial cells may ultimately determine its intracellular fate. Although internalization through Fc receptors delivers C. jejuni to lysosomes, when entering via its own specific adaptations C. jejuni segregates from the endocytic pathway and avoids delivery to lysosomes. The mechanisms of C. jejuni internalization are unusual in that they do not require the actin cytoskeleton and are dependent on microtubules [10]. In fact, disruption of the actin cytoskeleton increases the efficiency of bacterial uptake (Figure S6). Previous studies have shown that addition of filipin, an agent that sequesters cholesterol, decreased the ability of C. jejuni to enter into cultured epithelial cells [44,45], suggesting that lipid rafts or caveolae may be required for efficient entry into cells. Consistent with this observation, we found that addition of the cholesterol-sequestering agent methyl-beta cyclodextrin (MβCD) blocked C. jejuni internalization into T84 in a dose-dependent manner (Figure S6). Similar results were obtained with Cos-1 cells (data not shown). To further investigate the potential involvement of lipid rafts or caveolae in C. jejuni internalization, we examined the CCV for the acquisition of caveolin-1 and flotillin-1, two markers associated with these membrane domains [46–49]. Cos-1 cells were transfected with plasmids encoding GFP-tagged forms of caveolin-1 or flotillin-1, and the association of these markers with the CCV was examined by time lapse and fluorescence microscopy as described in Materials and Methods. C. jejuni acquired caveolin-1-GFP and flotillin-1-GFP immediately after internalization (Figure 7A and 7B). Quantification of this association determined that at early time points during infection, ∼60% of the CCVs colocalized with both caveolin-1-GFP (Figure 7C) and flotillin-1-GFP (Figure 7D and Video S2). The association, however, was transient since at later points after infection <10% of the CCVs were seen in association with these markers (Figure 7C and 7D). Vesicles devoid of bacteria but labeled by flotillin-1-GFP were also observed immediately after C. jejuni internalization, and some of them eventually fused with the nascent CCV (Video S3 and S4).

Caveolin-1 and flotillin-1 have been shown to be involved in various endocytic events, including the internalization of microbial pathogens [50]. We therefore further examined the potential involvement of caveolin-1 or flotillin-1 in C. jejuni internalization into non-phagocytic cells. Depletion of flotillin-1 by siRNA did not result in a measurable decrease in the abiliy of C. jejuni to enter cells (*p* = 0.13) (Figure S7). However, expression of a dominant interfering mutant of caveolin-1 (caveolin-1^Y14F^) significantly decreased C. jejuni internalization (*p* = 0.02) (Figure 7E). Taken together these data indicate that caveolin-stabilized lipid membrane domains (i.e., caveolae) are important for C. jejuni efficient entry into non-phagocytic cells.

The GTPase dynamin is involved in pinching off of the nascent endosome in both clathrin- and caveolae-mediated endocytosis [51–54]. We therefore tested the potential involvement of dynamin II in C. jejuni internalization. Cos-1 cells were transfected with a plasmid expressing a dominant negative form of dynamin II (dynII^K44A^), which has been shown to inhibit both clathrin and caveolae-dependent endocytosis [52–54], and the ability of C. jejuni to enter those cells was examined by fluorescence microscopy as described in Materials and Methods. Although expression of dynII^K44A^-GFP effectively blocked the uptake of transferrin (data not shown), it did not inhibit the uptake of C. jejuni (Figure 7F). In fact, there was a modest enhancement of C. jejuni internalization in the presence of wild-type dynamin II. These results indicate that the role of caveolin-1 C. jejuni entry into cells may not be related to its role in caveolae-mediated endoyctosis. Rather, caveolin-1 or caveolae may play a role in the signaling events leading to bacterial uptake, perhaps by facilitating the spatial organization of critical signaling molecules. In fact, tyrosine kinases have been reported to be essential for C. jejuni entry into host cells [44,45,55], a result that we have confirmed (Figure S6). Since efficient signaling through receptor tyrosine kinases is known to require lipid rafts or caveolae, it is possible that the inhibitory effect of cholesterol sequestering agents or dominant negative caveolin-1 may be the result of interference with tyrosine kinase signaling.

### The C. jejuni–Containing Vacuole Localizes in Close Proximity to the Golgi

**Figure 8:**
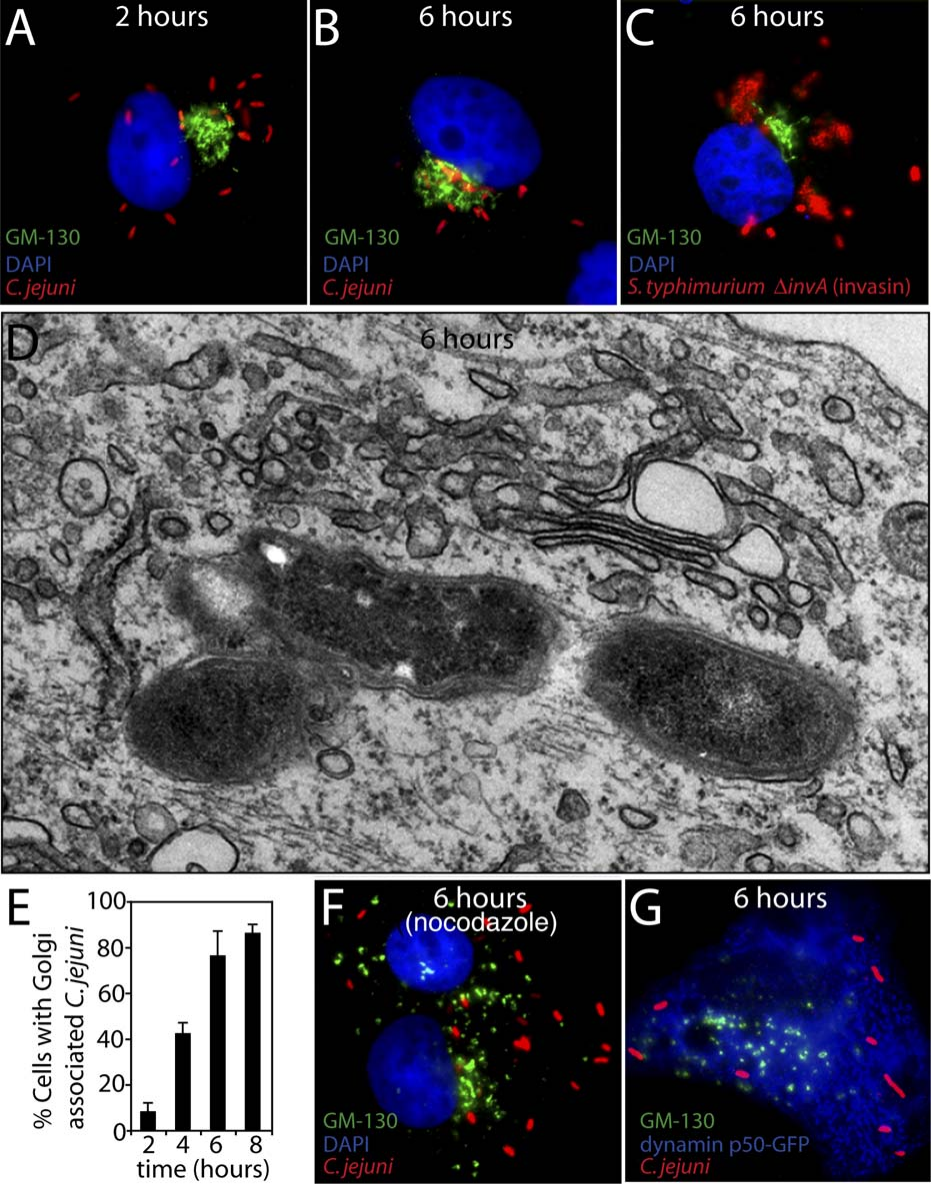
The C. jejuni–Containing Vacuole Localizes in Close Proximity to the Golgi Cos-1 cells were infected with C. jejuni or *S. typhimurium invA* (invasin) expressing dsRed for 1 h followed by a gentamicin chase. Cells were fixed at the designated times after infection and processed for immunofluorescence using anti-C. jejuni (red) and anti-GM-130 antibodies (green). Nuclei were visualized with DAPI (blue). Immunofluorescence images of *C. jejuni-*infected cells 2 h (A) and 6 h (B) post-infection, and *S. typhiumurium invA* (invasin)-infected cells 6 h post-infection (C). (D) Electron micrograph of C. jejuni-infected cells 6 h post-infection. (E) Quantitation of C. jejuni association with the Golgi at different times after infection. Values are averages and standard deviations of three independent experiments. A minimum of 100 infected cells were counted in each experiment (F) Nocodazole treatment prevents C. jejuni's close association with the Golgi. Cos-1 cells were infected with C. jejuni for 1 h and after a 1 h gentamicin chase, cells were treated with nocodazole for additional 4 h to disrupt microtubules. At 6 h after infection, cells were fixed and processed for immunofluorescence as described above. (G) Dymanin is required for the localization of C. jejuni in close association to the Golgi. Cos-1 cells were transfected with dynamatin p50-GFP and 24 h later, cells were infected with C. jejuni as described above. Six hours after infection, cells were fixed and processed for immunoflourecesce using anti-C. jejuni and anti GM-130 antibodies.

Examination of the localization of the CCV over time showed that at 4–5 h after infection, C. jejuni localized to the perinuclear region [56]. To gain more insight into the specific localization of the CCV in relation to other organelles, we investigated by immunoflurescence microscopy the position of the CCV in relation to the Golgi apparatus over time using an antibody directed against GM130, a Golgi resident protein. Two hours after infection, intracellular C. jejuni were evenly distributed around the cell (Figure 8A). However by 6–8 h of infection, >85% of the CCVs were seen in close association with the Golgi apparatus (Figure 8B and 8E), close to the microtubule organizing center (Figure S8). The close association of the CCV and the Golgi does not represent a default pathway for any internalized particle traveling to a perinuclear position since internalized *S. typhimurium invA* (invasin) did not show association to the Golgi despite the fact that these phagosomes were also located in a perinuclear region (Figure 8C). Electron microscopy analysis confirmed the intimate association of the CCV and the Golgi apparatus (Figure 8D). However, the CCV did not acquire Golgi markers (data not shown), indicating that despite their close association, the two compartments remain distinct.

We then investigated the mechanism by which the CCV reaches its perinuclear destination. Many intracellular bacteria in membrane-bound compartments traffic along microtubule tracks to reach their destination within the cell. We first investigated the role of microtubules in the localization of the CCV. When nocodazole was added to disrupt the microtubule network after infection of Cos-1 cells, the CCVs were observed distributed throughout the cell and did not reach a perinuclear location (Figure 8F). These data show that intact microtubules are necessary for the CCV to reach its final destination. Phagosomes most often move along microtubules through the action of specific motors [57,58]. Cytoplasmic dynein is a minus-end directed motor that is responsible for moving cargo away from the periphery and toward the microtubule organizing center [59] and is therefore a candidate motor to move the CCV to its final destination. We investigated this hypothesis by overexpressing GFP-dynamatin p50, a subunit of the dynactin complex that when overexpressed, blocks the function of dynein [60]. Immunofluorescence analysis showed that overexpression of dynamatin p50 effectively disrupts the localization of C. jejuni at 6 h post-infection (Figure 8G). These results are consistent with a previous observation indicating that addition of orthovanadate, a rather non-specific inhibitor of dynein, inhibits the movement of the CCV to a perinuclear position [56]. Taken together, these results indicate that subsequent to internalization, the CCV travels to a perinuclear position in the immediate vicinity of the Golgi apparatus and that the movement of the CCV requires both microtubules and the molecular motor dynein.

## Discussion

Similar to other enteric pathogens, C. jejuni has evolved the ability to gain intracellular access to non-phagocytic intestinal epithelial cells and this process has been implicated in pathogenesis [7,14,20,61,62]. Although most work to date has focused on C. jejuni entry into host cells, the intracellular fate of this pathogen has been largely uncharacterized. We have shown here that C. jejuni survives within intestinal epithelial cells, although over time it acquires a metabolic state that renders it unculturable under standard culture conditions. However, C. jejuni recovered from within epithelial cells could be cultured if subjected to conditions of severe oxygen limitation. These results suggest that once within epithelial cells, C. jejuni may either become oxygen sensitive or may alter its respiration mode so that it can no longer be cultured in the presence of oxygen. The recently completed nucleotide sequence of the genome of the C. jejuni strain 81–176 used in this study revealed the presence of genes involved in additional respiratory pathways, including electron acceptors that may be utilized for alternate modes of respiration [63]. Thus, these additional respiration genes may contribute to the ability of C. jejuni 81–176 to survive within intestinal epithelial cells.

**Figure 9:**
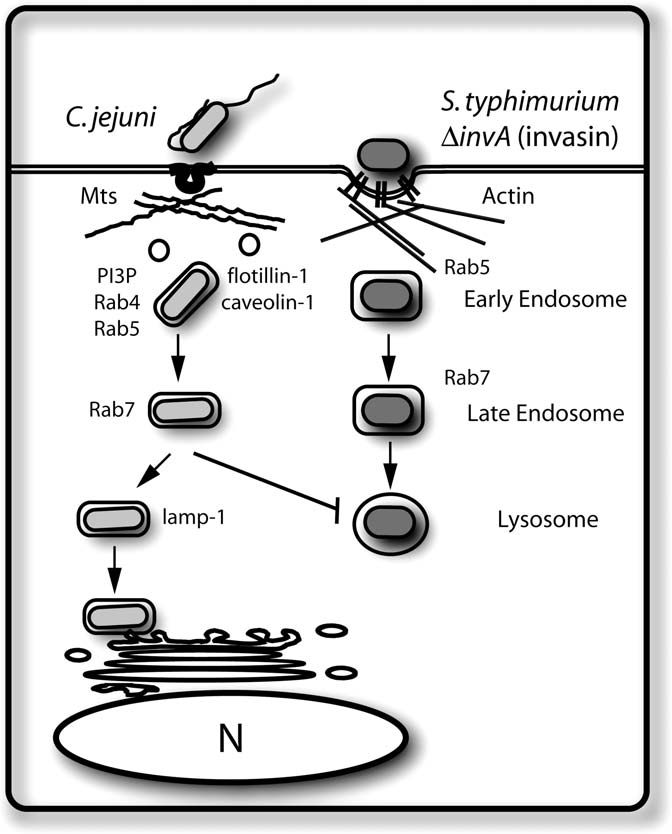
Model for C. jejuni Internalization and Trafficking within Epithelial Cells C. jejuni enters intestinal epithelial cell via a microtubule and caveolae-dependent process. After internalization, the C. jejuni–containing vacuole transiently acquires different markers of the endocytic pathway and ultimately survives within a compartment that is functionally separated from the canonical endocytic pathway, which is represented in this scheme by *S. typhimurium invA* (invasin).

We showed here that C. jejuni survives within intestinal epithelial cells within a compartment that is distinct from lysosomes (Figure 9). CCVs are not accessible to endocytic tracers indicating that they are functionally separated from described endocytic pathways leading to lysosomes. In fact, when targeted into lysosomes after internalization via the Fc receptor, C. jejuni was unable to survive within epithelial cells. These results indicate that C. jejuni has evolved specific adaptations to traffic within host cells and avoid delivery into lysosomes. These adaptations may be important to faciliate colonization of the host by providing a safe-heaven where C. jejuni can avoid innate immune defense mechanisms. However, those adaptations must not be able to operate in macrophages since, in these cells, C. jejuni is targeted to lysosomes and therefore cannot survive.

Our results suggest that C. jejuni deviates from the canonical endocytic pathway shortly after internalization (Figure 9). The CCV appears to interact with early endosomal compartments since it associates with early endosomal markers such as EEA-1, Rab5, Rab4, and PX-GFP (which labels PI3P). However, this interaction is transient and does not lead to progression within the canonical endocytic pathway. The presence of markers of lipid-associated rafts and caveolae on the CCV suggests that C. jejuni may reside in a compartment that is functionally distinct from early endosomes. In fact, C. jejuni was still able to target properly in the presence of dominant interfering mutants of Rab5 or Rab7, which control early events in the endocytic pathway [43].

C. jejuni has unusual cytoskeletal requirements to gain intracellular access to intestinal epithelial cells since its internalization is dependent on microtubules but not on the actin cytoskeleton [10], as is usually the case for most intracellular bacteria [22]. Consistent with previous observations suggesting that caveolae are required for C. jejuni entry [44,45], we have shown here that bacterial internalization is dependent on caveolin-1. However, we showed that the entry process is independent of dynamin, whose function is essential for clathrin and caveolae-mediated endocytosis [51–53]. In fact, expression of a dominant-inhibitory form of dynamin resulted in a reproducible increase in the ability of C. jejuni to enter cells. We therefore hypothesize that a caveolin-1-stabilized lipid membrane domain may be required for proper signaling through tyrosine kinases, which are also required for C. jejuni internalization rather than for endocytosis. In fact, efficient signaling through receptor tyrosine kinases requires lipid rafts or caveolae [64,65] and the surface availability of many these is regulated by dynamin [66]. In this context, we hypothesize that the enhancement of C. jejuni internalization observed when inhibiting dynamin function, may be the result of an increase in the availability of putative surface “receptor” for this pathogen resulting in enhanced signaling for entry.

What is the nature of the CCV? We showed that the CCV contains lamp-1, although this compartment is unique and clearly distinct from lysosomes since it does not colocalize with the lysosomal protein marker cathepsin B and it is not accessible to endocytic tracers. In fact, the acquisition of lamp-1, which occurs very early in the CCV maturation, must occur by an unusual mechanism that does not require the GTPases Rab5 or Rab7. Interestingly, S. typhimurium resides within a vacuole that is apparently segregated from the canonical endocytic pathway [27,67] and also harbors lamp-1, although in this case acquisition of this marker appears to require Rab7 [68]. Another unique property of the CCV is its close association with the Golgi, which requires microtubules and the motor protein dynein. More studies will be required to better define the nature of this compartment and the precise mechanisms by which C. jejuni modulates vesicular trafficking.

In summary, we have established that C. jejuni has evolved specific adaptations to survive within intestinal epithelial cells by avoiding delivery into lysosomes. This survival strategy does not appear to operate in BMDM since C. jejuni is rapidly killed in these cells. We hypothesize that C. jejuni's unusual entry mechanism may be central to its ability to avoid delivery into lysosomes since when internalized via a different pathway (e.g., via the Fc receptor), C. jejuni could not avoid delivery into lysosomes. Its diversion from a pathway leading to lysosomes must therefore occur upon entry. Understanding the mechanism by which this bacterium survives within host cells may provide new insights into C. jejuni pathogenesis as well as reveal undiscovered paradigms in host cellular trafficking.

## Materials and Methods

### Bacterial strains, culture conditions, and plasmids.

Wild-type C. jejuni 81–176 has been described previously [69]. C. jejuni were routinely grown on tryptic soy broth agar supplemented with 5% sheep blood (BA) or in brain heart infusion (BHI) broth at 37 °C under 10% CO_2_, or where indicated, in an anaerobic chamber under low oxygen conditions (BD-Diagnostic Systems GasPak Plus Anaerobic System Envelopes with Palladium Catalyst, catalog number 271040, New Jersey), or with BBL CampyPacks (BBL Microbiology Systems, Cockeysville, MD). *S. typhimurium invA* has been described previously [70] and was transformed with invasin-encoding plasmid pRI203, which mediates mammalian cell entry via αβ1 integrins [71]. A *S. typhimurium invA* strain expressing the dsRed protein was constructed as follows. The plasmid DsRed.T3_S4T, which expresses the dsRed protein under the control of an arabinose-inducible promoter [72], was digested with EcoRI and ScaI to release a fragment containing dsRed and the *paraABC* promoter. This fragment was ligated into pACYC184 and resulting plasmid, pSB3082, was transformed into *S. typhimurium invA* (pRI203). *S. typhimurium invA* expressing invasin and dsRed was routinely grown in LB containing tetracycline (10 μg ml^−1^), ampicillin (50μg ml^−1^), and 0.1% arabinose to induce DsRed expression.

### Cell culture and preparation of bone marrow–derived macrophages.

T84, a human intestinal epithelial cell line, and Cos-1, a monkey kidney epithelial cell line, were obtained from the American Type Culture Collection (Rockville, MD) and were grown in DMEM supplemented with 10% fetal bovine serum containing penicillin (100 U ml^−1^) and streptomycin (50 μg ml^−1^). Bone marrow–derived macrophages (BMDM), were obtained as previously described [73]. Briefly, femurs and tibias were excised and flushed with DMEM containing 10% fetal bovine serum (FBS), penicillin (100 U ml^−1^), and streptomycin (50 μg ml^−1^). Cells were spun down and resuspended in BMDM differentiation medium [DMEM containing 20% FBS, 30% L-cell supernatant, penicillin (100 U ml^−1^) and streptomycin (50 μg ml^−1^)] and plated onto non-tissue culture treated 10-cm^2^ plastic dishes. The cells were fed fresh BMDM differentiation medium on day 3–4 to allow further differentiation until day 6–7. BMDM were then seeded in the appropriate tissue culture dishes to be used in infection experiments.

### Bacterial infection of cultured cells.

C. jejuni was harvested from a fresh BA plate and grown in BHI broth under under 10% CO_2_ until mid log phase (OD_600_ = 0.7–0.8). To prepare the inoculum, bacteria were pelleted at 20,000 × g in a microfuge for 2 min and directly resuspended in Hank's Balanced Salt Solution (HBSS) (Invitrogen). The inoculum was diluted in HBSS to adjust for different multiplicity of infections (MOI). Serial dilutions of the inoculum were plated onto BA plates to determine the number of bacteria. T84 cells were split to 70% confluence (∼10^5^ cells per well) in a 24 well dish. BMDM were seeded at 2 × 10^5^ cells per well in a 24 wells dish. After washing 3X with HBSS, T84 cells and macrophages were infected with an MOI of 50 or 20, respectively. The plates were centrifuged at 200 × g for 5 min to maximize bacteria-cell contact and incubated for 1 or 2 h at 37 °C 5% CO_2_. Following the incubation, the monolayers were washed 3X with HBSS and incubated with complete media containing gentamicin (100 μg ml^−l^) for 2 h. This concentration of gentamicin was found to be optimal to kill extracellular bacteria without affecting the viability of intracellular bacteria. Plating of the infection medium determined that no significant number of c. f. u. were detected after this treatment. For experiments involving longer time points, the media was replaced with complete media containing gentamicin (10 μg ml^−1^). Again, plating of the infection medium determined that no significant number of c. f. u. were present after this treatment. After 3 additional washes, the infected cells were lysed at the designated time points and the samples were prepared for colony forming unit (CFU) determination or FACS analysis. To quantify the number of intracellular bacteria, cells were lysed in PBS with 0.1% deoxycholate and the CFU were enumerated after plating serial dilutions grown at 37°C with 10% CO_2_, or in GasPak jars (BBL Microbiology Systems, Cockeysville, MD) with BBL CampyPacks (BBL Microbiology Systems, Cockeysville, MD). For anaerobic incubations, plates were incubated in an anaerobic chamber with a GasPak (BD-Diagnostic Systems GasPak Plus-Anaerobic System Envelopes with Palladium Catalyst, Catalog number 271040, New Jersey) for 48 h and incubated further at 37 °C with 10% CO_2_. For immunofluorescence studies, Cos-1 cells were seeded on glass cover slips in 24 well plates. C. jejuni was cultured as described above. *S. typhimurium invA* expressing invasin was cultured in LB containing ampicillin (30 μg ml^−1^) overnight and subcultured 1:20 for 3 h prior to infection. For time courses, cells were infected with an MOI of 100 and 50 for C. jejuni and *S. typhimurium invA* (invasin), respectively, centrifuged from 5 min at 1,000 × g to maximize bacteria-host cell contact, and incubated for an additional 15 min at 37 °C 5% CO_2_. Wells were washed three times in PBS and either fixed in 4% PFA for a 15 min time point or the media was replaced with DMEM + 10% FBS with gentamicin (100 μg ml^−1^) to kill the extracellular bacteria and prevent additional bacterial internalization. At later time points cells were washed an additional three times in PBS, and fixed in 4% paraformaldehyde. After 4 h or longer infection times, no significant number of bacteria that had remained extracellular but attached to the cell were detected using the gentamicin treatment described (i.e., initial addition of 100 μg/ml for 2 h and subsequent addtion of 10μg/ml for the remainder of the experiment, see above) (Figure S9). For quantitation of intracellular bacteria in transfected cells, Cos-1 cells were infected with an MOI of 25 for 1 h as described above. Cells were washed and incubated for 2 additional hours in DMEM + 10% FBS with gentamicin (100 μg ml^−1^) to kill the extracellular bacteria and prevent additional bacterial internalization. The cells were then fixed and processed for immunofluorescence as described below.

### Inhibitors.

Where indicated, cells were incubated with Nocodazole (10 μM), Cytocholasin D (5 μM), Genistein (10 μM and 100 μM), dissolved in DMSO (Sigma), or methyl-beta cyclodextrin (MβCD) dissolved in PBS (1mM-10mM) 30 min prior to infection and kept throughout the duration of the incubation period. All control cells were treated with the appropriate solvent for the same length of time.

### Enumeration of C. jejuni in host cells by flow cytometry.

T84 cells and BMDM from wild-type mice were seeded at density of 10^5^ cells per well on a 24-well dish and infected with an MOI of 50 and 20, respectively. Following a 1 h incubation at 37 °C and 5% CO_2_, the cells were washed with HBSS and DMEM containing 10% FBS and 100 μg ml^−1^ gentamicin was added to each well. Cells were washed again and lysed at the designated time points in 500 μl of 0.05% sodium deoxycholate in PBS. The cell lysates were collected and subjected to a low speed spin (1,000 rpm) for 2 min to remove large cell debris. Supernatants were collected and intracellular bacteria were isolated by a 2 min high-speed spin (10,000 rpm). The isolated bacterial pellet was resuspended in 500 μl filter-sterilized staining buffer (PBS containing 1mM EDTA and 0.01% Tween). The bacteria were then stained with the reagents of a cell viability kit (BD Biosciences, San Jose, CA), which distinguishes live and dead cells by using a thiazole orange (TO) solution, which stains all bacteria, and propidium iodide (PI), which only stains dead bacteria. TO and PI were added to final concentrations 53 nM and 11 μM, respectively, in accordance with the manufacturer's instructions. After 5 min of staining, bacteria were pelleted, washed once in PBS, resuspended in 1 ml of PBS and analyzed by flow cytometry. The absolute count of live/dead bacteria was carried out by addition of 50 μl of a liquid suspension of a known number of fluorescent beads (supplied in the kit, BD Biosciences, San Jose, CA) following the manufacturer's instructions. Samples were analyzed on a FACS calibur flow cytometer. TO fluoresces primarily in FL1 and FL2; PI primarily in FL3. An SSC threshold was used, and cells and beads were gated using scatter and FL2, which detects the TO fluorescence and therefore the total bacterial population. In order to best discriminate between live and dead bacteria, an FL1 versus FL3 plot was used and live and dead populations were gated within this plot (dead cells, FL3+; live cells, FL1+). To determine the bacterial concentration, the following equation was used: # events in cell region/ # events in bead region x # beads/test/test volume × dilution factor = concentration of cell population. A plot was generated after using this equation to calculate the number of viable bacteria (in triplicate wells) in both T84 and BMDM at each time point.

### Immunofluorescence microscopy.

Cos-1 cells were infected as described above and at the designated time points, cells were washed three times in PBS and fixed in 4% paraformaldehyde (PFA) for 13 min at room temperature (RT). The fixed cells were washed three times in PBS and permeabilized by incubating them in PBS containing 3% non-fat milk and 0.05% saponin (PBS-MS) (Calbiochem). Cover slips were incubated in primary antibody diluted in PBS-MS for 30 min. The cover slips were then washed three times in PBS and incubated in secondary antibody. After two washes in PBS and two washes in deionized water, the cover slips were mounted onto glass slides using Prolong Gold antifade reagent (Molecular Probes). Images were acquired on a Nikon TGE2000-U Eclipse inverted microscope fitted with a Micromax Princeton digital camera controlled by the Metamorph software package, version 6.1 (Universal Imaging Corp., Downingtown, PA). When needed, inside-out staining was used to differentiate extracellular from intracellular bacteria. Briefly, before permeabilization with saponin, extracellular bacteria were stained with rabbit anti-C. jejuni antiserum in PBS containing 3% milk followed by Alexaflour 350-conjugated anti-rabbit antibodies (Molecular Probes). Cells were washed three times, permeabilized, and the total bacterial population was stained with rabbit anti-C. jejuni followed by Alexaflour 594-conjugated mouse anti-rabbit antibodies (Molecular Probes). After two washes in PBS and two washes in deionized water, the cover slips were mounted onto glass slides using Prolong Gold antifade reagent (Molecular Probes). Where indicated, nuclei stained with DAPI (Invitrogen).

### Antibodies.

Rabbit antibodies against C. jejuni were obtained by repeated immunization of rabbits with a mixture of equal amounts of formaldehyde and heat-killed whole cell C. jejuni. Anti-S. typhimurium (rabbit) antibodies were purchased from DIFCO Laboratories, Detroit, Michigan. Mouse monoclonal antibodies against EEA-1, Lamp-1, beta-tubulin, and GM130 were acquired from BD Biosciences Pharmingen. A mouse anti cathepsin B antibody was a gift from the laboratory of Dr. Ira Mellman, Yale University, New Haven, CT. Secondary antibodies used were: Alexa 596- Alexa-488, Alexa 350-conjugated goat anti-rabbit and Alexa 596- Alexa 488- Alexa 350-conjugated goat anti-mouse IgG antiserum (Molecular Probes).

### Plasmids and transient transfection.

Eukaryotic expression vectors encoding GFP-tagged wild-type Rab5 and Rab7 as well as their dominant negative mutants have been previously described [74–76]. GFP-tagged Rab4, PX, dynamin II, dynamin II^K44A^, caveolin-1, and caveolin-1^Y14F^ as well as murine Fc-receptorII (FcRII) expressing eukaryotic vectors have been described elsewhere [77–81]. The human flotillin-1 gene was amplified from a human cDNAs library by polymerase chain reaction (PCR) using primers fwd (5′-TAGCTCGAGCCATGTTTTTCACTTGTGGCCC-3′) and rev (5′-TCTAGAATTCCGGCTGTTCTCAAAGGCTTGA-3′). The PCR product was then cloned into pEGFP-N1 (Clontech, Oxford, UK) using XhoI and EcoR1. The resulting plasmid, pSB3111, yielded flotillin-1 fused to the N-terminus of GFP. pFlot1-FLAG was a generous gift from Dr. Rosanna Paciucci, Unitat de Recerca Biomedica, Barcelona, Spain [82]. Plasmid DNA was purified using the Maxiprep kit (Qiagen) and used for transfection of cells with LipofectAMINE 2000 (Invitrogen) according to the manufacturers instructions.

### Colocalization of markers with the CCV and bacterial quantitation of transfected cells.

To quantitate the percentage of CCV containing different endocytic fluid tracers or cellular markers, infected cells were visualized directly in the fluorescence microscope. Using the Metamorph software package a series of images were taken, including internalized bacteria, total bacteria, and the cellular marker. Overlayed fluorescent images were analyzed by determining the number of CCVs that contained the corresponding marker. A minimum of one hundred vacuoles was analyzed per cover slip for each treatment and designated post-infection time. Each experiment was completed in triplicate wells/cover slips and expressed as an average. CCVs were considered positive for the presence of a marker when they contained detectable amounts of the staining probe/antibody. The same methodology was used to quantitate the number of intracellular bacteria within transfected cells. Briefly, Cos-1 cells seeded on cover slips were transfected with plasmids encoding GFP-tagged proteins, infected, and processed for immunofluorescence. A minimum of 50 transfected cells were randomly selected and imaged as described above and the number of intracellular bacteria was quantitated within each cell. Each experiment was completed in triplicate wells/cover slips and expressed as an average number of bacteria per transfected cell and where designated normalized to control cells. Statistical analysis of the results was carried out by the student *t*-test.

### Fluid endocytic tracers and pulse-chase experiments.

Cos-1 cells were grown on coverslips in 24-well dishes and infected with C. jejuni or *S. typhimurium invA* (invasin) and dsRed at an MOI of 25 and 10 respectively. After spinning for 5 min at 200 × g and allowing the infection to proceed for 1 h, the cells were washed three times and fresh media containing gentamicin was added to kill the extracellular bacteria. After an additional 3 h the cells were incubated with Texas Red or Alexafluor-488 labeled dextran (1 mg/ml; Molecular Probes) for 1 h. The cells were washed three times and incubated with fresh media containing gentamicin for an additional 2 h. The cover slips were then processed for immunofluorescence.

### FcR-mediated C. jejuni internalization.

Cos-1 cells were plated in a 24 well dish at ∼40% confluency and transfected with a plasmid encoding FcRII [80]. After an overnight incubation, cells were incubated with C. jejuni that were opsonized with rabbit polyclonal anti-C. jejuni antibodies (1:1,000). Plates were centrifuged at 200 × g for 5 min and incubated at 37 °C 5% CO_2_ for 1 h, after which the cells were washed three times with HBSS, and incubated with complete media containing gentamicin (100 μg ml^−1^). Cells were then lysed at the designated time points and the number of viable intracellular bacteria was assessed as described above.

### Live cell imaging.

Cos-1 cells were grown on life cell imaging dishes (MatTek Corp.) and transfected with flotillin-1-GFP or PX-GFP [78] encoded eukaryotic expression vectors as described above. C. jejuni was cultured as described above and fluorescently labeled using PKH26 Red Flourescent Cell Linker Kit according to manufacturer's instructions (Sigma). The labeling protocol did not affect *C.jejuni*'s viability or its ability to enter into non-phagocytic cells (data not shown). Cells were infected with an MOI of ∼100. Time-lapse series were acquired using a Nikon TGE2000-U Eclipse inverted microscope fitted with a Micromax Princeton digital camera controlled by the Metamorph software package, version 6.1 (Universal Imaging Corp., Downingtown, PA). Filters allowed the simultaneous detection of GFP and rhodamnine, respectively. The acquired images were merged as RGBs (for two color movies) and converted into QuickTime movies using Metamorph. For the flotillin-1-GFP three dimensional movie, cells transfected with flotillin-1-GFP were infected for 15 min, fixed, and processed for imunoflourescence using inside-out staining. Z-stacks were acquired using a Zeiss Axio Imager Upright fluorescent Microscope fitted with an Apotome and the AxioCam MRc5 digital camera controlled by the Axiovision software package, version 4.2 (Carl Ziess MicroImaging, Inc.). The cropped images were reconstructed and converted into a 3D QuickTime movie using Axiovision.

### Gene silencing by RNAi.

Depletion of flotillin-1 was performed using a pool of three target-specific 20–25 nucleotide siRNAs designed to knock down gene expression (Santa Cruz Biotechnology, Inc., Santa Cruz, CA). The siRNA pool was transfected into Cos-1 cells using LipfectAMINE 2000 (Invitrogen). To mark transfected cells, flotillin siRNA was cotransfected with pEGFP-N1 at a ratio of 5:1. Intracellular bacteria within GFP-expressing cells were quantified as described above. RNAi silencing efficiency and specificity were analyzed at the protein level by Western blot analysis 72 h after cotransfection of pFlot1-FLAG [82] with the siRNA pool. Protein depletion by RNAi was normalized to endogenous levels of actin using rabbit anti-actin antibodies (Sigma-Aldrich).

### Electron microscopy.

Cos-1 cells or BMDM were plated at approximately 8 × 10^6^ cells on 10 cm^2^ plastic tissue culture dishes. After 3x wash with warm HBSS, C. jejuni, cultured as described above, were used to infect cells at an MOI of 100. At 1.5 h post-infection, the extracellular bacteria were washed off three times with warmed HBSS and the plates were incubated with warmed culture media containing gentamicin to kill the extracellular bacteria. For standard EM, the infected cells were incubated at 37 °C 5% CO_2_ for and additional 4.5 h. For BSA-gold experiments, after a 3.5 h incubation, the infected cells were incubated for 1 h with BSA-gold-containing complete media. After three additional washes cells were fixed in situ with a freshly made solution of 1% glutaraldehyde (from an 8% stock from Electron Microscopy Sciences (EMS), Fort Washington, PA) 1% OsO_4_ in 0.05 M phosphate buffer at pH 6.2 for 45 min. After fixation, the cells in petri plates were rinsed three times with cold distilled water and en bloc stained with uranyl acetate overnight. The petri plates were then dehydrated in ethanol then placed into hydroxypropyl methacrylate (EMS), which does not react with the plastic in the petri dish, and embedded in L 112, an epon substitute (Ladd, Burlington, VT). Following polymerization of the epon, the block was cut out and mounted and thin sections were cut through their exposed surfaces. Thin sections were collected on naked grids stained with uranyl acetate and lead citrate and examined in a Philips 200 electron microscope. At least fifty vacuoles were analyzed for the presence of BSA-gold for each condition.

## Supporting Information

Figure S1 The C. jejuni–Containing Vacuole Is Not Accessible to the Endocytic Tracer Dextran (5.0 MB TIF)

Figure S2 Acquisition of EEA-1 and lamp-1 by the C. jejuni–Containing Vacuole (4.2 MB TIF)

Figure S3 The C. jejuni–Containing Vacuole Acquires Markers of the Early Endocytic Pathway (8.3 MB TIF)

Figure S4 The C. jejuni–Containing Vacuole Acquires Rab7 but Not Cathepsin B (8.2 MB TIF)

Figure S5 Dominant-Negative Rab5 and Rab7 Do Not Block Acquisition of lamp-1 by the C. jejuni–Containing Vacuole (11.9 MB TIF)

Figure S6 C. jejuni Internalization into Cultured Intestinal Epithelial Cells in the Presence of Different Host Cell Inhibitors (11.9 MB TIF)

Figure S7 Flotillin-1 Is Not Essential for C. jejuni Internalization into Epithelial Cells (2.0 MB TIF)

Figure S8 Intracellular Campylobacter jejuni Localizes in Close Proximity to the Microtubule-Organizing Center (926 KB TIF)

Figure S9 Absence of Extracellular Bacteria after Gentamicin Treatment (415 KB TIF)

Video S1 C. jejuni Recruitment of PX-GFP (1.2 MB MOV)

Video S2 Colocalization of C. jejuni with Flotillin-1-GFP (489 KB MOV)

Video S3 C. jejuni Recruitment of Flotillin-GFP (2.6 MB MOV)

Video S4 Flotillin-1-GFP Dynamics during C. jejuni Infection (3.4 MB MOV)
