# Supplementary figures and images for: Campylobacter jejuni Survives within Epithelial Cells by Avoiding Delivery to Lysosomes
Source: PLoS Pathog. 2008 Jan 25;4(1):e14. doi: 10.1371/journal.ppat.0040014 (PMC2323279; doi:10.1371/journal.ppat.0040014)

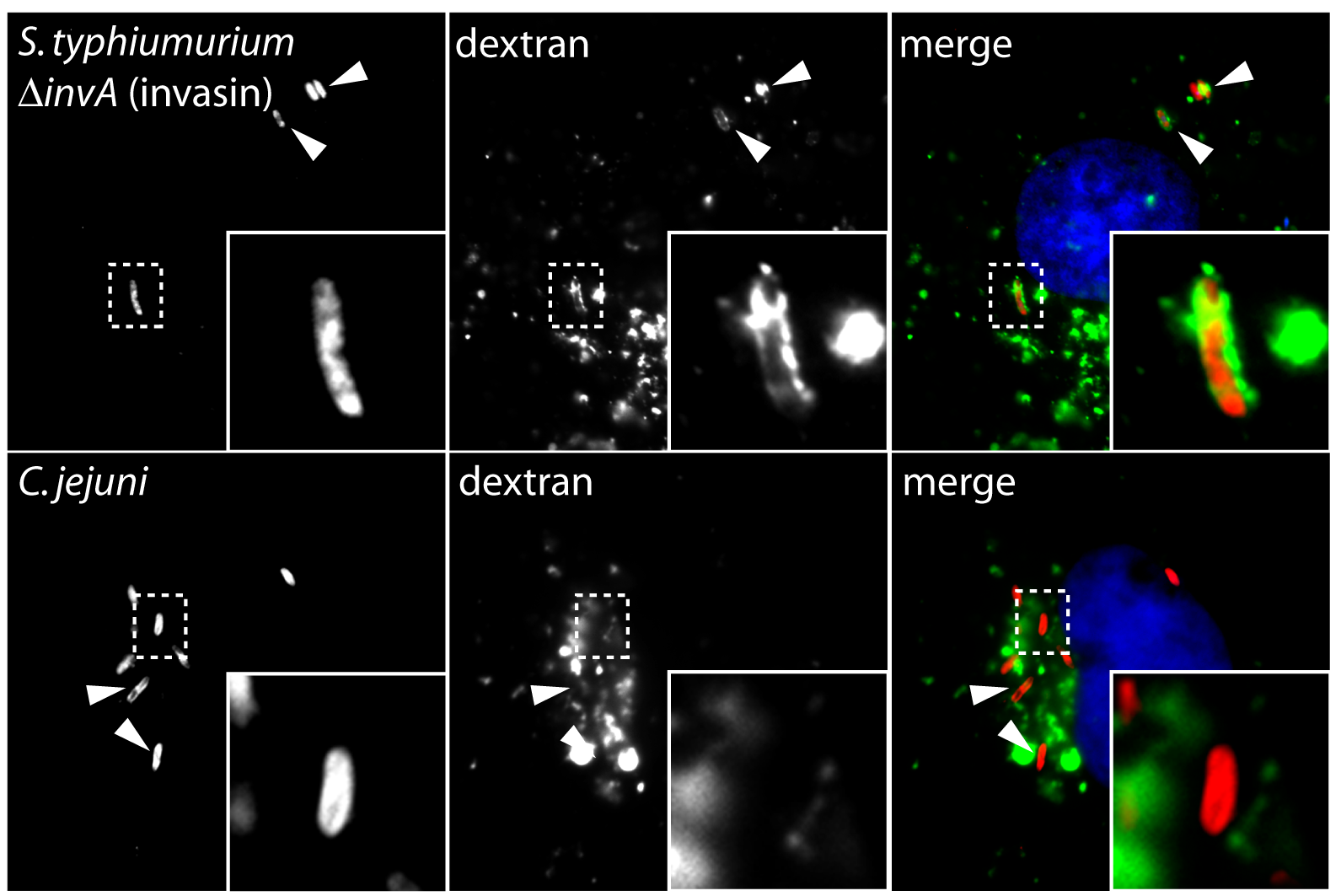

Supplement: Figure S1 — (5.0 MB TIF) [file ppat.0040014.sg001.tif]

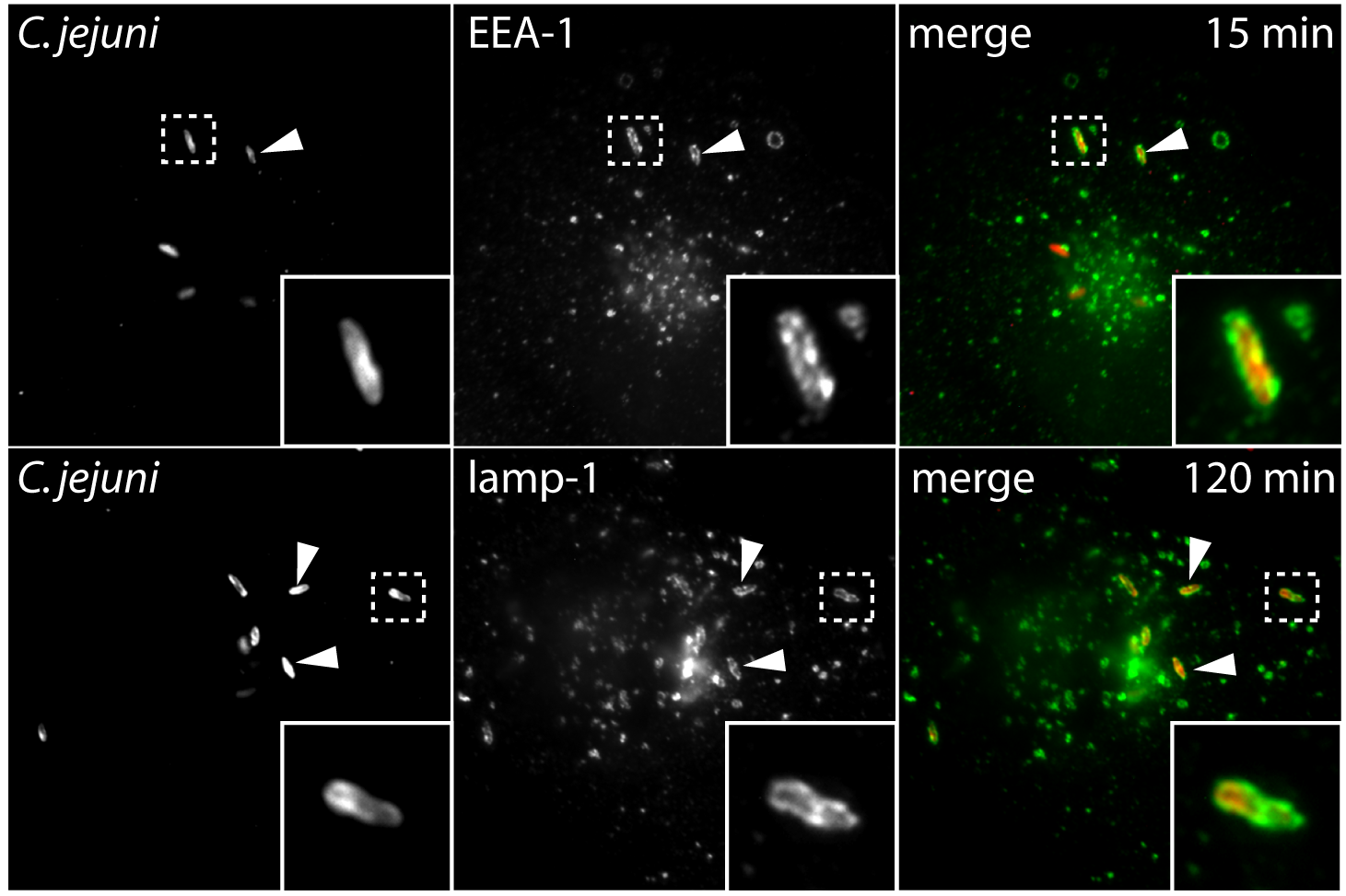

Supplement: Figure S2 — (4.2 MB TIF) [file ppat.0040014.sg002.tif]

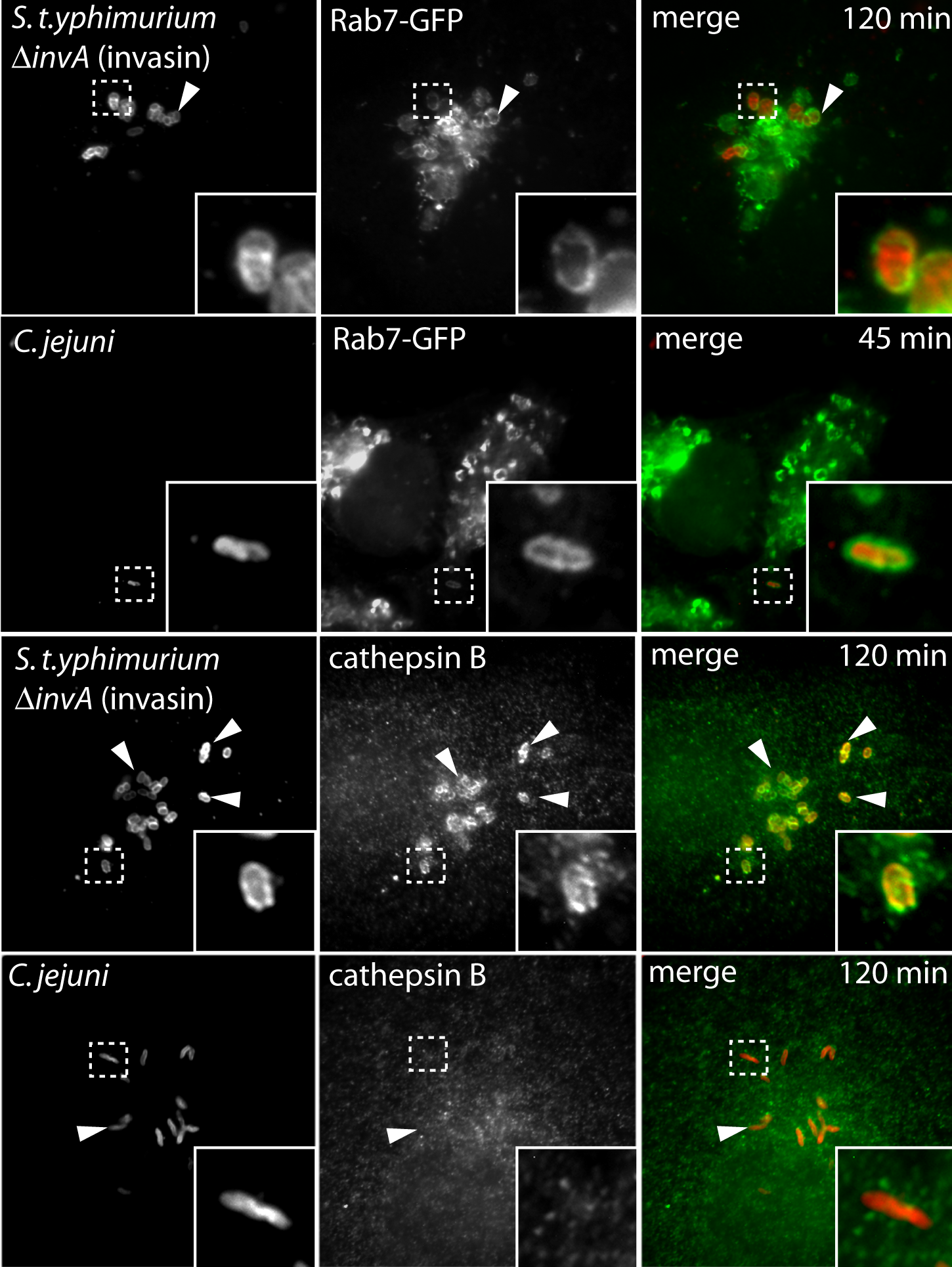

Supplement: Figure S4 — (8.2 MB TIF) [file ppat.0040014.sg004.tif]

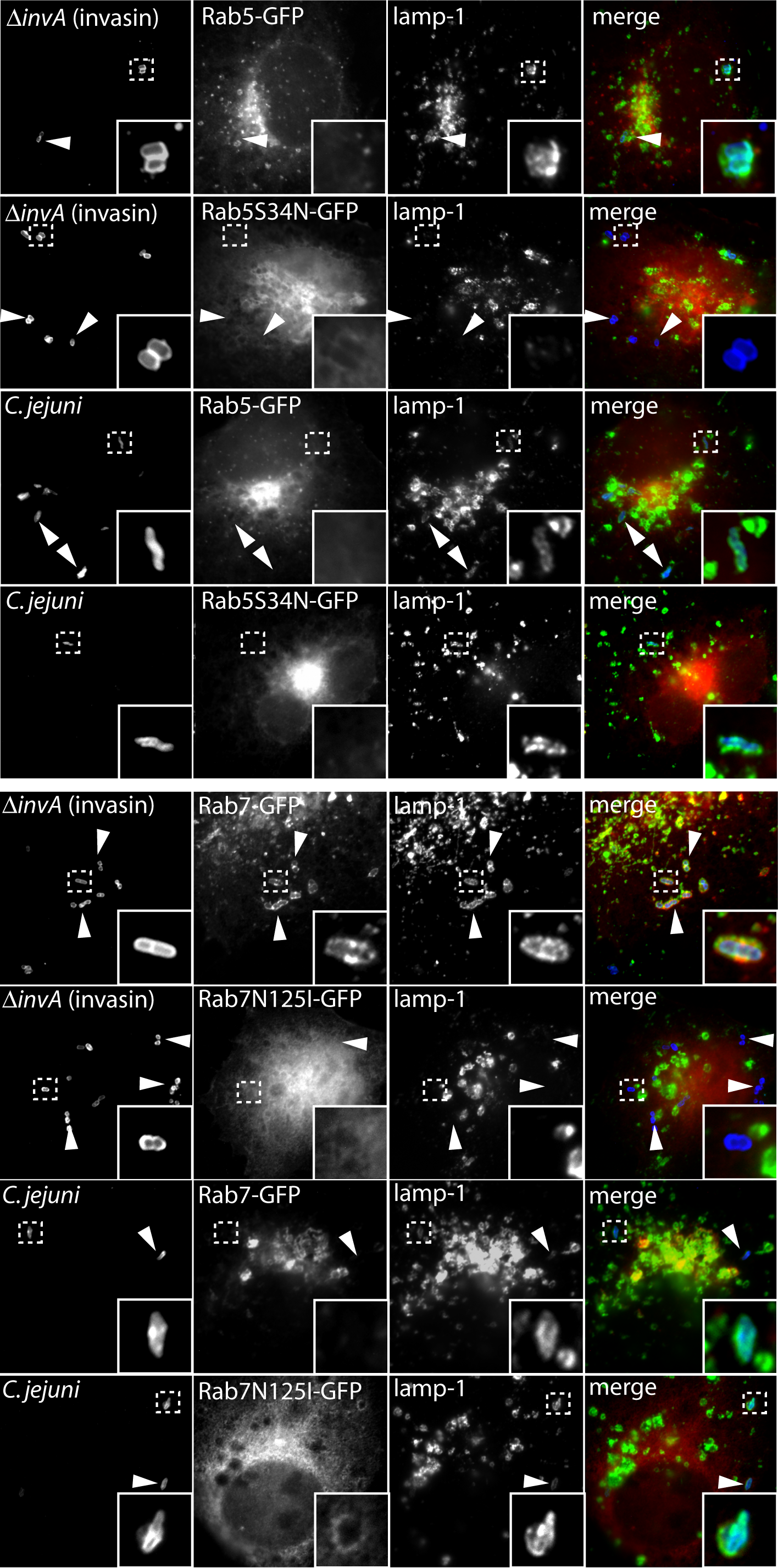

Supplement: Figure S5 — (11.9 MB TIF) [file ppat.0040014.sg005.tif]

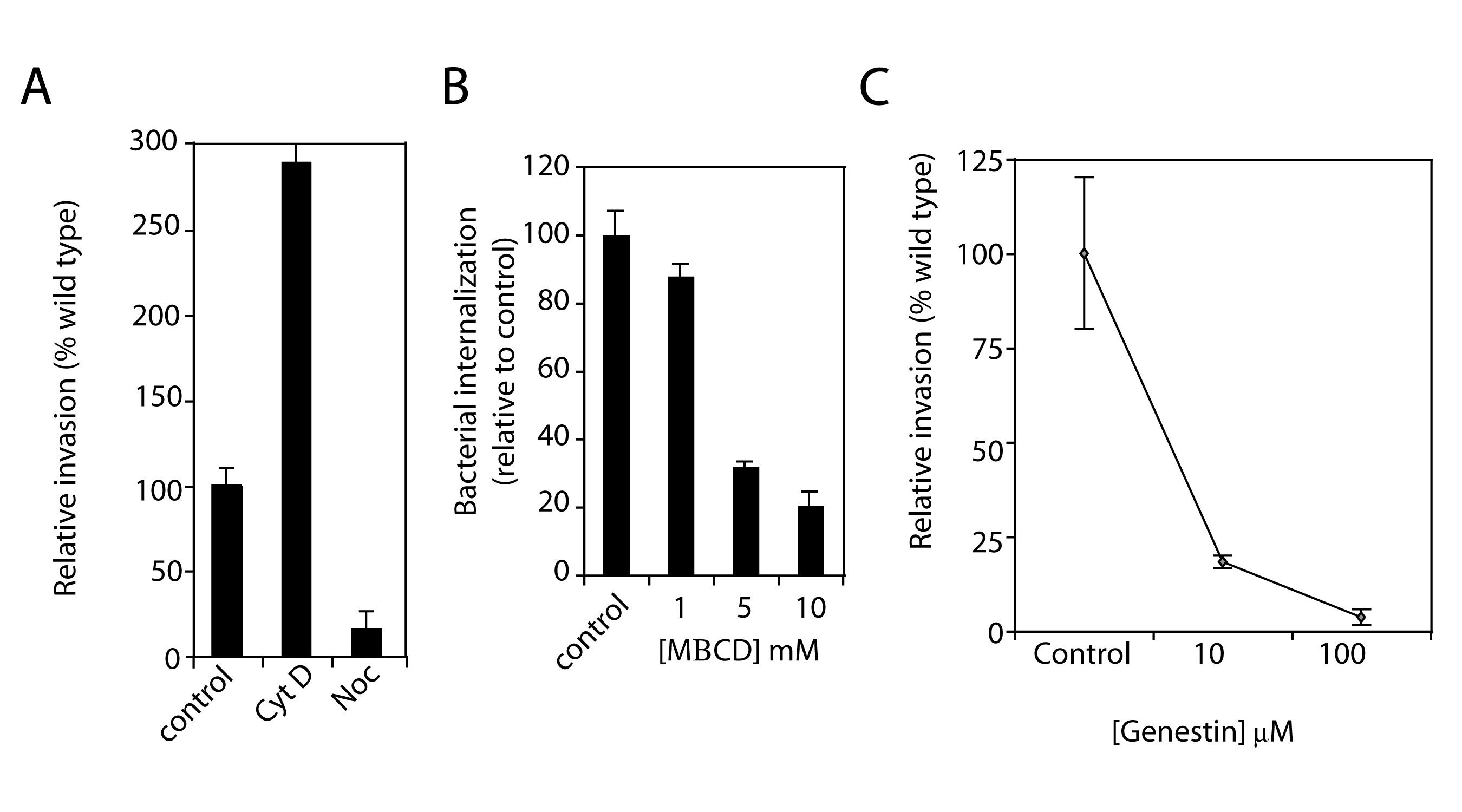

Supplement: Figure S6 — (11.9 MB TIF) [file ppat.0040014.sg006.tif]

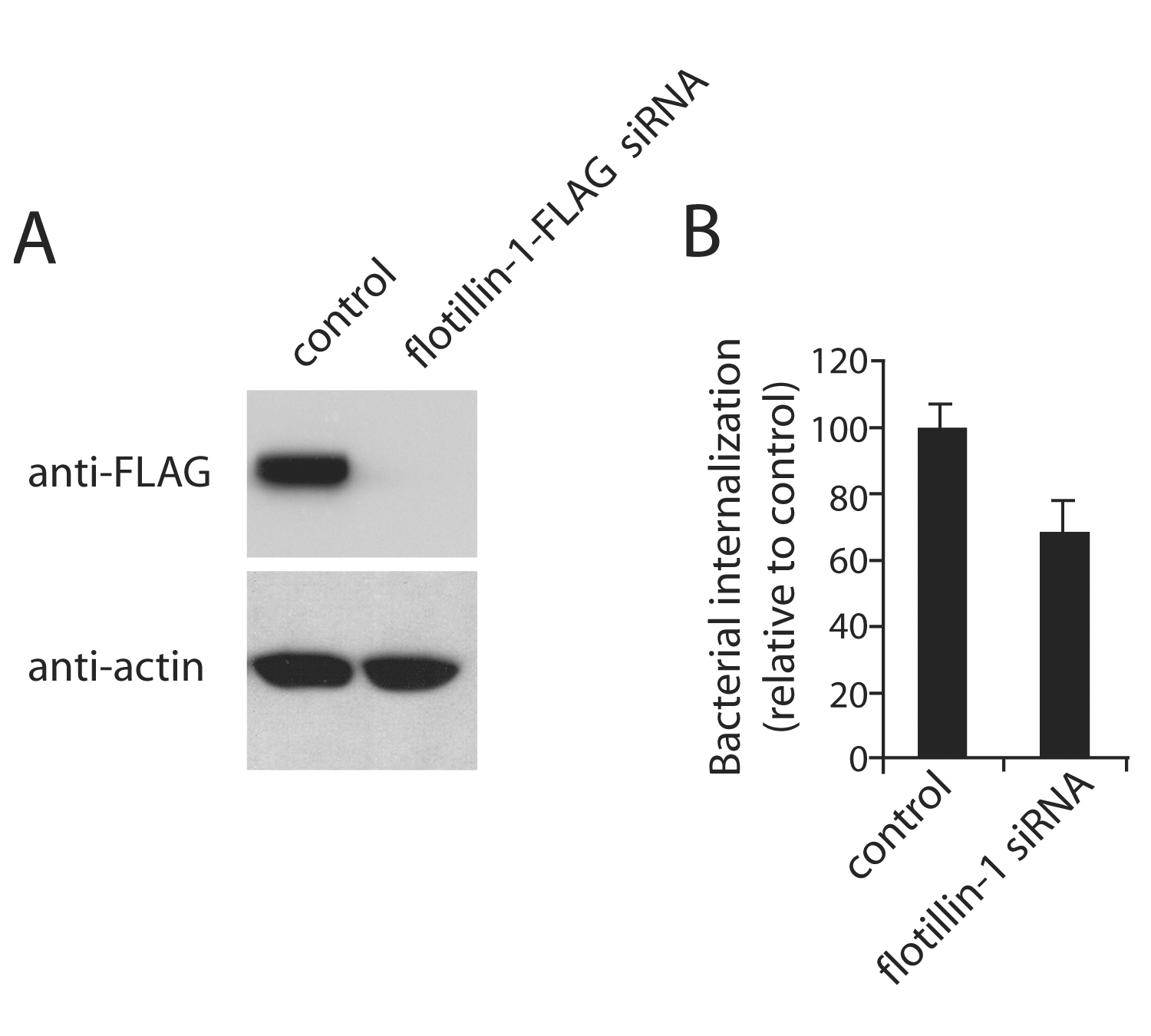

Supplement: Figure S7 — (2.0 MB TIF) [file ppat.0040014.sg007.tif]

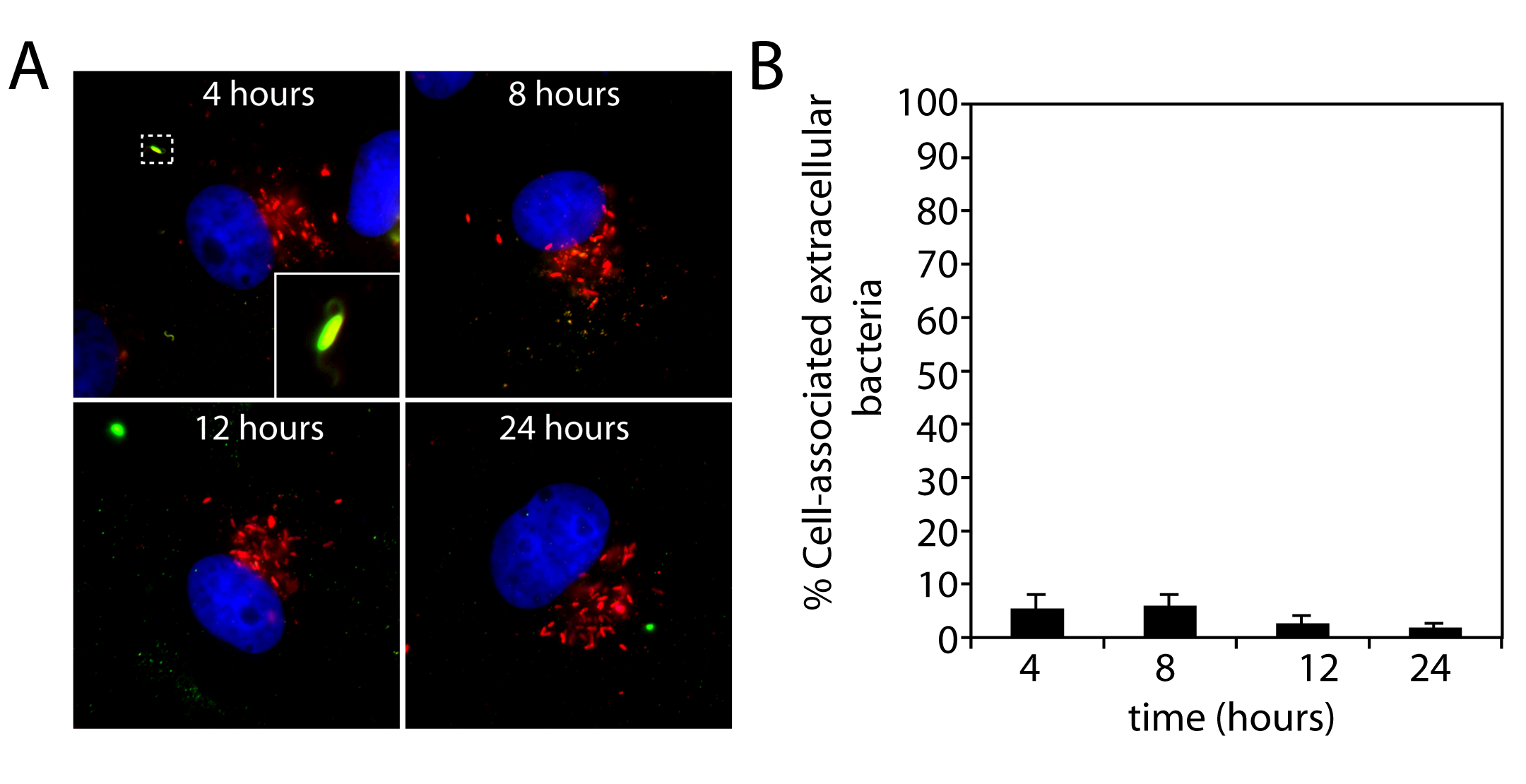

Supplement: Figure S9 — (415 KB TIF) [file ppat.0040014.sg009.tif]
